# Vancomycin in infusion during vitrectomy in surgical treatment of acute postoperative and posttraumatic endophthalmitis

**DOI:** 10.1186/s12879-016-1830-6

**Published:** 2016-09-20

**Authors:** Robert Rejdak, Tomasz Choragiewicz, Agnieszka Kalinowska, Michael J. Koss, Piotr Ksiazek, Joanna Moneta-Wielgos, Ryszard Maciejewski, Anselm G. Jünemann, Katarzyna Nowomiejska

**Affiliations:** 1 Department of General Ophthalmology, Medical University of Lublin, ul. Chmielna 1, 20-079 Lublin, Poland; 2 Department of Experimental Pharmacology, Medical Research Centre, Polish Academy of Sciences, Warsaw, Poland; 3 Department of Didactics and Medical Simulation, Human Anatomy Chair, Medical University of Lublin, Lublin, Poland; 4 Department of Ophthalmology, University of Heidelberg, Heidelberg, Germany; 5 Department of Public Health, Medical University of Lublin, Lublin, Poland; 6 Human Anatomy Department, Medical University of Lublin, Lublin, Poland; 7 Department of Ophthalmology, University of Rostock, Rostock, Germany

**Keywords:** Endophthalmitis, Ocular trauma, Vitrectomy

## Abstract

**Background:**

Endophthalmitis is potentially devastating intraocular inflammation following eye trauma or surgery. We describe the visual outcomes and causative pathogens in acute bacterial postoperative and posttraumatic endophthalmitis treated with immediate pars plana vitrectomy (PPV) with Vancomycin dissolved in the infusion fluid.

**Methods:**

Clinical records of consecutive 30 patients with postoperative endophthalmitis and 15 patients with posttraumatic endophthalmitis were evaluated. Vancomycin was administered constantly in the infusion fluid at the time of complete PPV. Cultures were prepared from anterior chamber paracentesis. The mean follow-up period was 13 months.

**Results:**

The visual acuities were improved in 38 cases (84 %) and remained stable in seven cases (16 %). Median post-PPV visual acuity was 1.0 logMAR in a group with postoperative endophthalmitis and 1.3 logMAR in a group with posttraumatic endophthalmitis (*p* < 0.05). Twenty cases (44 %) were culture-positive (*Staphylococcus, Streptococcus, Enterococcus* and *Bacillus spp*).

**Conclusions:**

Early PPV with Vanomycin in infusion leads to vision improvement in patients with both posttraumatic and postoperative endophthalmitis. In our series of 45 cases culture was positive only in half of the cases.

## Background

Endophthalmitis is a severe and potentially devastating intraocular purulent inflammation resulting from infectious agent invasion to the posterior segment of the eye occurring as a complication of an operation procedure, penetrating eye injuries or hematogenous spread of bacteria [1]. Endophthalmitis may lead to loss of vision due to irreversible damage of photoreceptors caused by bacterial toxins.

Approximately 70 % of cases occur as a complication of the intraocular surgery, mostly cataract extraction. Capsular or zonular surgical complication and wound leak on the next day after surgery are the main risk factors for the postoperative endophthalmitis [2]. Posttraumatic endophthalmitis following open-globe injuries accounts for one-fourth of all cases of endophthalmitis [3] and may have a significant impact on socioeconomic life. The likelihood of infection after penetrating ocular trauma (3.3 to 17 %) is 100 times greater than after cataract surgery (0.05 %) [4]. Rural setting [5], delayed wound closure [6] and dirty wound [7] are the main risk factors for endophthalmitis after penetrating eye trauma. Other risk factors are: retained intraocular foreign body (IOFB) [6] and ruptured lens capsule [4].

The treatment of endophthalmitis has evolved during the last several decades. Before the age of vitreoretinal surgery, enucleation of affected eye was usually performed [8]. In the 1970s treatment of endophthalmitis greatly improved by introduction of intravitreal antibiotics. Intravitreal injection of empirical choice antibiotic (1 mg Vancomycin or 2.25 mg Ceftizidime) is still used in many countries in the primary treatment of endophthalmitis and is called the “silver standard” by European Society of Cataract and Refractive Surgeons (ESCRS) guidelines [2]. Since 1980, the success rate of surgical treatment-pars plana vitrectomy (PPV)–defined as final vision of 20/400 or better–has increasingly improved, ranging from 42 to 73 %. Therefore, complete PPV is now considered the “gold standard” in the treatment of endophthalmitis [2].

Vancomycin is considered to be the drug of choice for empiric coverage of Gram-positive organisms – the most common cause both in posttraumatic and postoperative endophthalmitis. Vancomycin is widely used in the treatment of endophthalmitis as intravitreal injection alone or at the end of PPV using the 25G or 30G needle [2].

The objective of this study was to characterize the visual outcome and causative pathogens in patients with postoperative and posttraumatic endophthalmitis who had been treated with immediate PPV with Vancomycin dissolved in infusion.

## Methods

In this noncomparative case series we have analyzed retrospectively medical records of consecutive 45 patients with acute endophthalmitis - 15 posttraumatic and 30 acute postoperative bacterial endophthalmitis treated at the Department of General Ophthalmology in Lublin, Poland, between 2010 and 2015. This study followed the tenets of the Declaration of Helsinki. The treatment chosen in the study was a part of a standard care. The study was approved by the local Ethic Committee at the University in Lublin, Poland. The patients gave their written informed consent to participate in the study and publish the data.

Inclusion criteria were as follows: (1) clinical signs of endophthalmitis: lid edema, chemosis, conjunctival hyperaemia, anterior chamber and vitreous cells, hypopyon, corneal edema, corneal ring infiltrate, reduced red reflex and afferent pupillary defect; (2) eye trauma or intraocular surgery performed less than 6 weeks ago; (3) PPV with Vancomycin in tapping performed within 6 h from the beginning of signs of endophthalmitis.

The diagnosis of endophthalmitis was made on the basis of clinical examination and ultrasonography. The patient data were recorded including: age, gender, type of trauma, previous surgical procedures, primary and final visual acuity, result of microbiological examination. Functional (best-corrected visual acuity: improved, stable, worsened) and anatomic outcomes (retinal attachment, intraocular pressure) were assessed during the follow-up.

Snellen decimal best corrected visual acuity was recorded in the affected eyes and converted into logarithm of the minimum angle of resolution (logMAR). Initial and the final best-corrected visual acuities were compared.

There were 13 men and two women in posttraumatic group and 14 men and 16 women in postoperative group. Overall, the median age of patients was 60 years (range 20–80 years), 40 years in posttraumatic patients and 70 years in postoperative patients (*p* < 0.0001).

Previous surgical procedures included: 20 phacoemulsyfications with IOL implantation, three bevacizumab injections, three secondary IOL implantations, one trabeculectomy, one Ahmed valve implantation, one PPV, one extracapsular cataract extraction.

Most of the surgeries included cataract surgery. As all cataract surgeries were performed in another hospitals, we do not have a knowledge what was the incision length, if preoperative fluorochinolones and intracameral cefuroxime was used as a prophylaxis of endophthalmitis.

Ocular trauma data included the mechanism and type of injury classified according to the Ocular Trauma Classification. Zone I injuries are confined to the cornea, zone II injuries involve the anterior 5 mm of the sclera, and zone III injuries involved full-thickness scleral defects more posterior than 5 mm from the limbus [9]. Wound location in posttraumatic eyes was classified as zone I in seven eyes, as zone II in three eyes and as zone III in three eyes.

The preoperative examination included visual acuity, intraocular pressure and B-scan ultrasonography. The follow-up examination included: visual acuity, intraocular pressure, and retinal attachment assessed with fundus examination after dilatation. The mean follow-up period was 13 months (range 10–20 months). Patients were examined on the next day after 1 week, after 1 month, 3 months, 6 months and year after operation.

Statistical computations were performed using GraphPad InStat software.

### Surgical procedure

At the day of admission (within 6 h) a standard three-port immediate and complete 23G PPV (Constellation, Alcon, US) was performed under general or peribulbar anesthesia. In posttraumatic cases the corneal or scleral laceration was sutured, if it was not done in the region hospital.

**Fig. 1:**
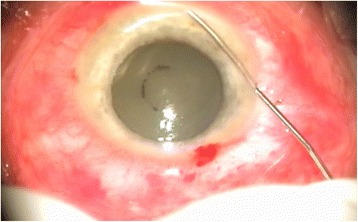
Aspiration of the hypopyon for microbiological culture

Microbiological cultures were prepared from anterior chamber via paracentesis (Fig. 1) or using the vitrectomy cutter before switching on the irrigation. To obtain anterior-chamber samples 27G needle was inserted into the anterior chamber via limbus and 0.1 mL of fluid was aspirated to the tuberculin syringe. On the initiation of the PPV 0.2 mL of undiluted vitreous was aspirated into a syringe connected to the infusion line.

**Fig. 2:**
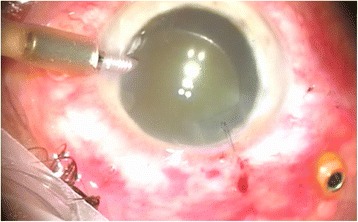
Securing anterior chamber maintainer

**Fig. 3:**
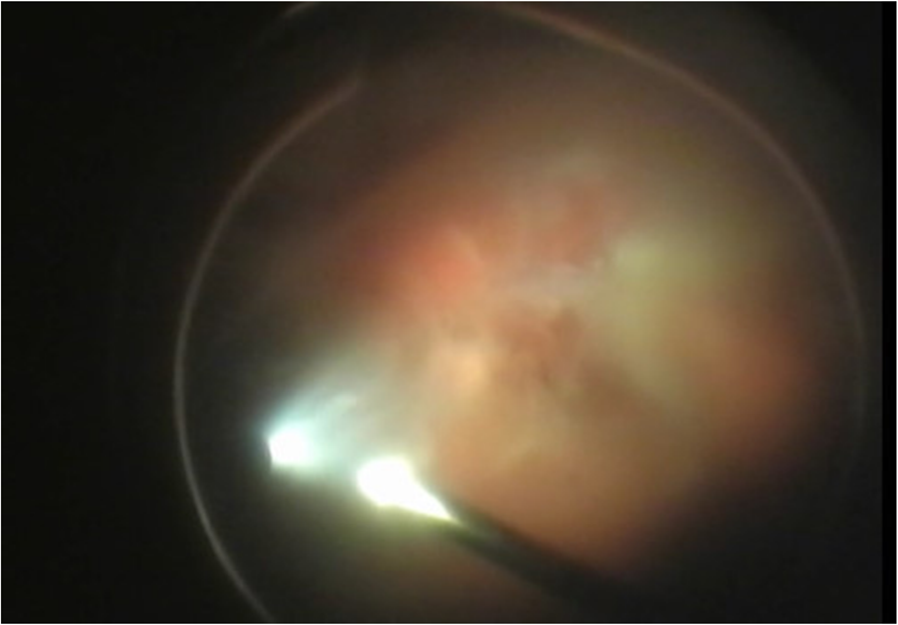
Posterior vitreous detachment during vitrectomy

**Fig. 4:**
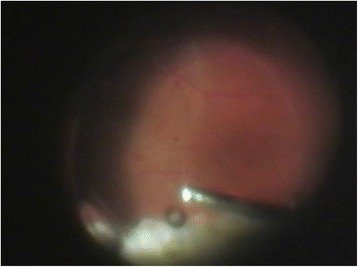
Shaving peripheral vitreous during vitrectomy

After phacoemulsification with intraocular lens (IOL) implantation in phakic eyes was done, the anterior chamber maintainer was installed (Fig. 2) if there were difficulties with visualization of the infusion line into vitreous cavity. Next, the induction of posterior vitreous detachment (Fig. 3) as well as periphery shaving (Fig. 4) was performed. Intraocular Vancomycin (0.2 mg/ml) was administered at the time of PPV in the balanced salt solution (BSS) Plus (Alcon, Forth Worth, Texas, US) of the infusion line and irrigated continuously during the surgery. Vancomycin solution was prepared in the operation theatre by dissolving 1 g of Vancomycin in 10 ml of sterile normal (0.9 %) sialine and then dissolving 1 ml of this mixture in a sterile bottle of 500 ml of infusion fluid. The cost of one cruet containing 1 g of Vancomycin is about 2 euro. This cruet is bought by the hospital pharmacy in advance.

If aspirate was to be cultured, the material suctioned with the use of the instrument was collected in a sterile bottle. A positive culture was interpreted as either growth of the identical organism on two or more media or confluent growth on one medium at the inoculation site [10]. All patients received topical (moxifloxacin-every 1 h) and intravenous (cephazolin–2 x 1 g per day) broad-spectrum antibiotics to cover both Gram-positive and Gram-negative bacteria. This treatment was empirical and administered before the culture result was known.

## Results

Study criteria were met by 45 patients: 30 with postoperative endophthalmitis and 15 with posttraumatic endophthalmitis (Tables 1 and 2). Median preoperative visual acuity in group with postoperative endophthalmitis was 3.0 logMAR, post PPV – 1.0 logMAR (*p* = 0.0001). Median preoperative visual acuity in group with posttraumatic endophthalmitis was 3.0 logMAR, post PPV – 1.3 logMAR (*p* = 0.0001). There were no sinificant differences between final visual acuities between groups (*p* < 0.05).

**Table 1:** Patient data, previous surgical procedures, surgical procedure in the treatment of endophthalmitis and results of visual acuity and result of microbiological culture regarding group of 30 patients with postoperative endophthalmitis

No	Gender, age (years)	Previous surgical procedure	Surgical procedure, tamponade	Initial visual acuity logMAR	Final visual acuity logMAR	Result of microbiological culture
1	Male, age 50–55	Phaco + IOL, trabeculectomy	PPV + BSS	3.0	2.0	Culture negative
2	Male, age 70–75	Extracapsular cataract extraction, aphakia	PPV + silicone oil	3.0	2.0	*Culture negativee*
3	Male, age 80–85	Phaco + IOL,	PPV + silicone oil	3.0	1.0	Culture negative
4	Female, age 70–75	Phaco + IOL	PPV + silicone oil	3.0	3.0	*Enterococcus fecalis*
5	Female, age 80–85	Phaco + IOL	PPV + BSS	3.0	2.0	Culture negative
6	Male age 70–75	Avastin injection	Phaco + IOL+ PPV + BSS	1.3	1.3	*Staphylococcus epidermidis*
7	Male, age 55–60	Secondary IOL implantation	PPV + BSS	3.0	0.4	*Staphylococcus epidermidis*
8	Male, age 70–75	Phaco + IOL	PPV + BSS	3.0	1.0	Culture negative
9	Female, age 80–85	Phaco + IOL	PPV + BSS	3.0	1.0	*Staphylococcus aureus*
10	Female, age 70–75	Secondary IOL implantation	PPV + BSS	3.0	0.7	Culture negative
11	Male, age 50–55	Phaco + IOL + PPV + silicone oil	PPV + silicone oil removal + BSS	1.3	1.0	*Staphylococcus epidermidis*
12	Female, age 65–70	Ahmed valve implantation, Phaco + IOL	PPV + KPL + TKP + IOL explantation + silicone oil	3.0	3.0	Culture negative
13	Female, age 60–65	Phaco + IOL	PPV + BSS	3.0	1.0	Culture negative
14	Female, age 65–70	Phaco + IOL	PPV + silicone oil	3.0	1.0	*Staphylococcus aureus*
15	Female, age 80–85	Phaco + IOL	PPV + silicone oil	3.0	1.3	*Staphylococcus aureus*
16	Male, age 75–80	Avastin intravitreal injection	Paco + IOL + BSS	3.0	1.0	Culture negative
17	Male, age 80–85	Phaco + IOL	PPV + BSS	3.0	0.4	Culture negative
18	Male, age 70–75	Phaco + IOL	PPV + BSS	3.0	0.7	*Staphylococcus epidermidis*
19	Male, age 65–70	Avastin intravitreal injection	PPV + phaco + IOL + silicone oil	3.0	1.3	*Staphylococcus epidemidis*
20	Female, age 70–75	Phaco + IOL	PPV + silicone oil	0.7	0.5	Culture negative
21	Male, age 20–25	Phaco + IOL	PPV + silicone oil	2.4	0.4	Culture negative
22	Male, age 50–55	Phaco + IOL	PPV + silicone oil	3.0	1.0	*Staphylococcus epidermidis*
23	Female, age 70–75	Secondary IOL implantation	PPV + BSS	2.4	2.0	Culture negative
24	Female, age 60–65	Phaco + IOL	PPV + silicone oil	2.4	1.0	Culture negative
25	Female, age 70–73	Phaco + IOL	PPV + silicone oil	2.4	2.4	*Enterococcus fecalis*
26	Male, age 70–75	Phaco + IOL	PPV + BSS	2.4	0.7	Culture negative
27	Female, age 80–85	Phaco+ IOL	PPV + BSS	3.0	3.0	*Streptococcus oralis*
28	Female, age 80–85	Phaco + IOL	PPV + silicone oil	2.4	1.7	Culture negative
29	Female, age 55–60	Phaco + IOL	PPV + silicone oil	2.1	2.4	*Bacillus cereus*
30	Female, age 60–65	Phaco + IOL	TPPV + BSS	2.1	0.7	Culture negative

*Abbreviations*: *Phaco* phacoemulsification, *PPV* pars plana vitrectomy, *BSS* balanced salt solution, *IOL* intraocular lens, *KPL* keratoplasty, *TKP* temporary keratoprosthesis

**Table 2:** Patient data, type of injury, surgical procedure and results of visual acuity and result of microbiological culture examination regarding group of 15 patients with posttraumatic endophthalmitis

No	Gender, age	Type of injury, zone	Surgical procedure, tamponade	Initial visual acuity logMAR	Final visual acuity logMAR	Result of microbiological culture
1	Female, age 75–80	Pseudophakic, zone I	PPV + BSS	2.0	0.3	*Staphylococcus aureus*
2	Male, age 45–50	IOFB, traumatic cataract, zone I	PPV + phaco + IOL + silicone oil	3.0	2.4	Culture negative
3	Male, age 25–30	IOFB, traumatic cataract zone I	PPV + phaco + IOL + silicone oil	3.0	2.0	Culture negative
4	Male, age 55–60	Pseudophakic, zone III	PPV + BSS	3.0	0.4	*Staphylococcus aureus*
5	Male, age 45–50	Pseudophakic, zone I	PPV + BSS	2.0	1.3	Culture negative
6	Male, age 55–60	Traumatic cataract, zone III	PPV + phaco + IOL+ silicone oil	3.0	1.3	Culture negative
7	Female, age 35–40	Traumatic cataract, zone I	Phaco + IOL + KPL + PPV + silicone oil	3.0	3.0	*Bacillus cereus*
8	Male, age 40–45	IOFB, traumatic cataract, zone I	PPV+ phaco + IOL+ silicone oil	2.0	2.0	Culture negative
9	Male, age 20–25	Traumatic cataract, retinal detachment, vitrous haemorrhage, IOFB, zone II	PPV+ phaco + IOL+ silicone oil	3.0	3.0	*Bacillus cereus*
10	Male, age 30–35	Traumatic cataract, zone III	PPV + phaco + IOL++silicone oil	3.0	0.7	Culture negative
11	Male, age 60–65	Traumatic cataract, IOFB, zone II	PPV + phaco + IOL + silicone oil	3.0	2.0	*Staphylococcus aureus*
12	Male, age 55–60	Traumatic cataract, retinal detachment, IOFB, zone II	PPV+ phaco + IOL+ silicone oil	3.0	2.0	*Staphylococcus epidermidis*
13	Male, age 30–40	IOFB, traumatic cataract	PPV+ phaco + IOL + BSS	3.0	0.5	Culture negative
14	Male, age 20–30	Traumatic cataract, zone I	PPV+ phaco + IOL silicone oil	3.0	1.0	*Staphylococcus epidermidis*
15	Male, age 30–40	IOFB, traumatic cataract	PPV+ phaco + IOL SF6 gas	3.0	0.5	Culture negative

*Abbreviations*: *Phaco* phacoemulsification, *PPV* pars plana vitrectomy, *BSS* balanced salt solution, *IOL* intraocular lens, *IOFB* intraocular foreign body, *TKP* temporary keratoprosthesis

Overall, after PPV the visual acuities were improved in 38 cases (84 %) – 12 posttraumatic and 26 postoperative, stable in seven cases (16 %) cases - 3 posttraumatic and four postoperative.

Mean intraocular pressure was 14 mmHg (range 7–18 mmHg) before treatment and 19 mmHg (range 15–24 mmHg) after follow-up period.

In 12 cases PPV was combined with traumatic cataract removal with IOL implantation. Eyes after Avastin injections were phakic and PPV was combined with phacoemulsification and IOL implantation, in one case PPV was combined with IOL explantation. Retinal detachment was present in two cases of posttraumatic eyes, IOFB was present in eight cases. The mean time from the injury to wound repair was 2 days (1–4 days).

As a tamponade, BBS (20 cases - 4 posttraumatic and 16 postoperative) or 5000 sct silicone oil (25 cases–10 posttraumatic and 19 postoperative) or SF6 gas (one posttraumatic case) was used. PPV was combined with temporary keratoprosthesis (TKP) and keratoplasty in two patients (one posttraumatic and one postoperative).

Positive culture from either anterior chamber paracentesis or vitreous tapping or both were found in seven cases of posttraumatic eyes (47 %) of 15 patients (5 - *Staphylococcus epidermidis;* 2 *- Bacillus cereus*) and in 13 (43 %) cases of postoperative endophthalmitis (6 - *Staphylococcus epidermidis,* 3 - *Staphylococcus aureus*, 2 - *Enterococcus fecalis*, 1 - *Bacillus cereus 1 - Streptococcus oralis*). In 17 postoperative and eight posttraumatic cases culture was negative.

In the follow-up period of 1 year no retinal detachment was observed. Silicone oil was removed after mean period of 1 year and all retinas were attached. One from the operated cases, which underwent PPV combined with TKP and penetrating keratoplasty due to postoperative endophthalmitis following Ahmed valve implantation, ended in the phthisis bulbi. No eye was enucleated, no eye revealed signs of sympathetic ophthalmia.

## Discussion

In the present study we retrospectively evaluated data of patients with endophthalmitis following intraocular surgery and open globe eye trauma treated with immediate 23G PPV with Vancomycin dissolved in the infusion fluid.

The Endophthalmitis Vitrectomy Study (EVS) – the major randomized trial in this subject - published in 1995 recommended intravitreal antibiotics if the visual acuity is greater than hand motion and PPV if visual acuity is equal to light perception [11]. EVS was designed to compare vitreous tapping with vitrectomy only in the treatment of postoperative endophthalmitis following cataract surgery and secondary IOL implantation. In this study there was no difference between final visual acuities with and without the use of systemic antibiotics. In EVS study 100 % of Gram-postive bacteria were susceptible to Vancomycin.EVS study showed that immediate (defined by EVS as within 6 h from the presentation) PPV has satisfactory results and quicker recovery of vision in the management of this severe ocular infection only in eyes presented with light perception. However, many of the patients treated with vitreous tapping in fact underwent a mechanical vitrectomy, which is also called a vitreous biopsy. In effect, many of these patients underwent a vitrectomy, but it was a subtotal procedure (about 50 % of the vitreous) without induction of posterior vitreous detachment.

In our study all the patients underwent a “complete” PPV with Vancomycin dissolved in the infusion fluid and there was significant improvement of the visual acuity both in postoperative and posttraumatic endophthalmitis. As it was advocated by Kuhn and co-workers, withdrawal of the vitreous body during PPV removes the bulk of infectious organisms and associated inflammatory debris load in the vitreous cavity and provides a large quantity of specimen for diagnostic smear and culture, and enables better antibiotics distribution [12]. Moreover, PPV reduces the incidence and severity of retinal, especially macular, complications and thus achieves a higher possibility of favorable visual outcome. Early PPV seems to be much more preferred in posttraumatic endophthalmitis, as more profound inflammation is observed and there is increased probability of more virulent organisms.

The surgical technique of PPV in endophthalmitis is similar to a standard PPV with some exceptions. Securing the infusion cannula correctly may be difficult due to purulent vitreous body and hazy cornea. A longer infusion cannula (6 mm) or anterior chamber maintainer may be useful. If here is oedema of the corneal epithelium, its scraping may be necessary to obtain good visibility of the retina and vitreous. In most of the cases removal of the hypopyon and fibrous membranes form the anterior chamber using 23G forceps is necessary. Peripheral shaving should be performed with care to avid iatrogenic breaks of the retina, as adherent vitreous and fragile retina may be more prone to developing retinal breaks and detachments. Posterior vitreous detachment was induced in all cases. Many surgeons give Vancomycin intravitreally at the end of the surgery, however, in our opinion, applying Vancomycin instantly during PPV in the infusion line gives the highest concentration “at the target site” and seems to be better for the circulation of the antibiotic in the eye during surgery (about 1 h). Intravitreal administration of Vancomycin lasts for the limited time period only, there is a risk of iatrogenic damage – cataract and retinal breaks. Moreover, the margin for error between chemotherapy and retinal toxicity is very narrow.

Vancomycin is effective against most Gram-positive bacteria, also methycillin-resistant, including *Staphylococcus, Streptococcus* and *Bacillus* species. Vancomycin is a glycopeptide antibiotic inhibiting bacterial cell wall protein synthesis by binding to the 30S rRNA molecule of the bacterial ribosome. Resistant mutants are very rare. Vancomycin seems to be well tolerated by ocular structures, it is cleared from the eye anteriorly, resulting in a long half-life. There is also no evidence of retinal toxicity. Data from rabbit studies demonstrated a vitreal half-life of 20–40 h in uninfected rabbit eyes and 38–54 h in infected rabbit eyes [13].

It has been shown that intravenous Vancomycin does not reach therapeutic concentrations in vitreous due to the protective effect of the blood-ocular fluid barrier [14]. However, intraocular inflammation increases the permeability of the blood-ocular fluid barrier, enhancing penetration of systemic antibiotics into the vitreous cavity [14].

There are no strict indications for intravitreal Vancomycin injection as the primary treatment. It depends on the individual decision made by the surgeon. In our opinion it should be reserved for endophthalmitis cases with visible red reflex and visual acuity better than 0.1.

In a rabbit model of endophthalmitis caused by intravitreal injection of *Bacillus cereus*, it has been demonstrated that eyes treated with combined PPV and intraocular Vancomycin during the early stage of infection resulted in significantly greater retinal function compared with that of intraocular antibiotics alone [15].

There are reports that Vancomycin in some countries, especially in Europe, is used intracamerally or in the irrigating solution during cataract surgery as a prophylaxis of endophthalmitis [16, 17]. However, it may increase the risk of Vancomycin-resistant bacteria growth, thus Vancomycin should not be used for prohylaxis of endophthalmitis.

Direct intracameral injection of cefuroxime (1 mg) given at the end of the surgery is recommended by the ESCRS guidelines as a standard prophylactic intervention significantly reducing the postoperative infections rate [2]. In our hospital we use intracameral cefuroxime routinely in all patients at the end of cataract surgery, without exceptions, but we are not confident if it is the same in all regional hospitals in Poland. In a recent national Survey in Europe it was investigated the role of intracameral cefuroxime in the prevention of endophthalmitis following cataract surgery [18]. An incidence of endophthalmitis greater than 0.13 % was encountered significantly more frequently among centers that did not employ intracameral cefuroxime. However, we need to have updated data and try to choose the antibiotics based on the local microbiological surveillance [18]. Preoperative application of povidone - iodine to skin and conjunctiva seems to be proven intraoperative endophthalmitis prophylaxis [19]. This is the best surgical preparation technique. The systemic antibiotics decrease the bacteria loading to the eye in endophthalmitis. In our group of patients we used intravenous cephazolin - a first generation cephalosporin antibiotic. Topical antibiotic used in this study was fluorochinolon – moxifloxacin. It has been shown that fuorchinolones penetrate into the inflamed and non-inflammed vitreous better than other classes of antibiotics [20]. However, in the light of “choosing wisely” philosophy we should not probably use fluorquinolones preoperatively, but we also think that in the real world medical legal issues may predominate in our decisions [21]. Large studies have shown that there are several changes in bacteria composition of the conjunctiva over time [22–24]. Moreover, our concerns are about the increased resistance of Gram + germs to fluorquinolones. From the other side, it was proven that postoperative usage of fluorquinolones reduces the risk of endophthalmtis [25].

It is difficult to compare our results with other studies, as there is no standardized approach to the management of posttraumatic endophthalmitis. Microincision vitrectomy surgery, including 23- and 25-gauge PPV, is often described as being minimally invasive.

In our study most of the cases were treated with 23G PPV and silicone oil as a tamponade, as it does not support microbial growth [26]. Moreover, in an in vitro study, silicone oil was effective in the suppression of multiple endophthalmitis causing organisms [27]. The technological advances of small-gauge PPV seem to afford visual benefit, with more rapid healing, less discomfort, and an acceptably low incidence of adverse events compared with those observed in conventional 20-gauge PPV [28]. A discussion still exists concerning the appropriate timing for PPV in traumatized eyes. However, most reports agree that PPV should be performed without delay in severe cases of endophthalmitis, especially those involving IOFB [6].

In the present study there were no significant differences in final visual acuity between eyes with postoperative and posttraumatic endophthalmitis. However, it has already been shown, that there are different factors responsible for worse than postoperative endophthalmitis visual prognosis: virulence of the infectious organism, the severity of associated trauma, the rapidity of diagnosis and institution of therapy [29], as well as poor presenting visual acuity, inability to visualize the optic disc on indirect ophthalmoscopy, presence of vitreous membranes on ultrasonography, trauma by a needle and a culture-positive vitreous biopsy [3].

In our study the positive cultures were found in 44 % of cases. In the literature the rate of positive cultures is similar: 40 % in study of 97 patients by Vedantham [30], 44 % in the study of 955 samples analyzed by Ramakrishnan and co-operators [31] and 58 % in the study of 62 patients conducted by Das [32].

There has been wide variety of pathogens found in the studies dealing with posttraumatic and postoperative endophthalmitis [32]. Coagulase-negative *Staphylococcus* remains the most frequently identified cause of endophthalmitis [33]. *Bacillus cereus* is ranked second behind *Staphylococci* in the prevalence of posttraumatic endophthalmitis and some cases are polymicrobal. In our study *Staphylococcus epidermidis*, *Bacillus cereus*, *Enterococcus fecalis* and *Streptococcus oralis* were found in microbiological examination in half of cases. The low rate of culture positivity in this study could be due to poor sampling technique, the use of antibiotics postoperatively or simply sterile endophthalmitis. It has been already reported that cases with negative cultures at presentation were more likely to have improvement in visual acuity compared with culture-positive cases [34]. Ramakrishnan and colleagues [31] reported in 10-years retrospective study that the intraocular specimen may remain culture-negative despite obvious signs of infection in certain cases of posttraumatic endophthalmitis.

In the setting of an IOFB or soil contamination there is a high incidence of *Bacillus species* endophthalmitis [6]. Endophthalmitis caused by *Bacillus cereus* is characterized by a rapid onset of severe pain, inflammation and progression to panophthalmitis. The current antibiotic treatment for acute-onset bacterial endophthalmitis includes Vancomycin for gram-positive coverage and either ceftazidime or an aminoglycoside for gram-negative coverage [35].

The incidence of posttraumatic endophthalmitis may be decreased by early wound closure and prompt initiation of antibiotics. Prophylactic factors in the setting of trauma include primary wound repair within 24 h, lack of tissue prolapse into wounds and self-sealing wounds [36]. Systemic broad-spectrum antibiotic therapy is a common approach to prophylaxis against endophthalmitis in the setting of open-globe injury [37].

## Conclusions

We believe that Vancomycin is still an excellent antibiotic for the empirical treatment of bacterial endophthalmitis. Antibiotic choice in preventing endophthalmitis after ophthalmic surgery should be based on knowing the organisms causing endophthalmitis in that region, along with their antibiotic susceptibilities. In every tertiary vitreoretinal center, clear treatment guidelines should be established, all necessary antibiotics and an examination of the microbiological specimens should be available at all times.

The results of this retrospective study support the use of early PPV combined with Vancomycin in infusion in cases of both postoperative and posttraumatic infectious endophthalmitis. With this strategy the final visual outcome may be markedly improved.
